# Molecular characterization and phylogenetic placement of a new cestode, *Oochoristica turpanoeremiadis* sp. nov. (Cestoda: Cyclophyllidea: Linstowiidae), parasitizing *Eremias roborowskii* from Turpan Basin in China

**DOI:** 10.1017/S0031182026102315

**Published:** 2026-07

**Authors:** Xiaofei Yan, Shengtao Chen, Ziqi Jiang, Chong Liu, Yi Ba, Yiyang Zhao, Shuxian Li, Bin Zheng, Yafang Xu, Qing Han

**Affiliations:** 1 Xinjiang Agricultural University, Xinjiang Key Laboratory for Ecological Adaptation and Evolution of Extreme Environment Biology, College of Life Sciences, China; 2 College of Veterinary Medicine, Xinjiang Agricultural University, China; 3 National Center for International Research on Tropical Diseases, National Institute of Parasitic Diseases, Chinese Center for Disease Control and Prevention (Chinese Center for Tropical Diseases Research), National Health Commission Key Laboratory of Parasite and Vector Biology, WHO Collaborating Center for Tropical Diseases, China

**Keywords:** comparative morphology, *Eremias roborowskii*, integrative taxonomy, molecular phylogenetics, *Oochoristica*

## Abstract

The genus *Oochoristica* Lühe, 1898 (Cyclophyllidea: Linstowiidae) primarily parasitizes reptiles. A new species of *Oochoristica* was collected from the small intestine of *Eremias roborowskii* (Bedriaga, 1906), from the Turpan Basin of the Xinjiang Uygur Autonomous Region, China. Following hematoxylin staining, through microscopic examination and detailed morphological description, a comparative morphometric analysis was conducted against closely related congeners, with particular emphasis on key taxonomic indicators, including the reproductive system and cephalic structures. Phylogenetic relationships were inferred using a multilocus analysis based on the *18S* rRNA, *ITS1-5.8S-ITS2* and *COI* gene regions. Genetic distances were calculated to support species delimitation. The parasite exhibits the following diagnostic features: proglottids significantly wider than long; a scolex lacking a rostellum and hooks; 20–25 spherical testes clustered in the posterior region of the proglottid; genital pores irregularly alternating along the lateral margins; a bilobed, highly branched ovary; and an elliptical vitellarium situated anterior to the ovary. Phylogenetic trees demonstrated that the new species formed a distinct, well-supported clade with lower intraspecific genetic variation than interspecific divergence, thereby supporting its status as an independent species. This new species is the 19th *Oochoristica* taxon recorded in the Palaearctic realm and the first novel cestode found in *Eremias roborowskii*. In the present study, an integrative taxonomic approach was employed, which combined comparative morphology with molecular phylogenetics and zoogeographic analyses, thereby elucidating the phylogenetic significance of this species within the genus *Oochoristica*.

## Graphical abstract

**Figure:**
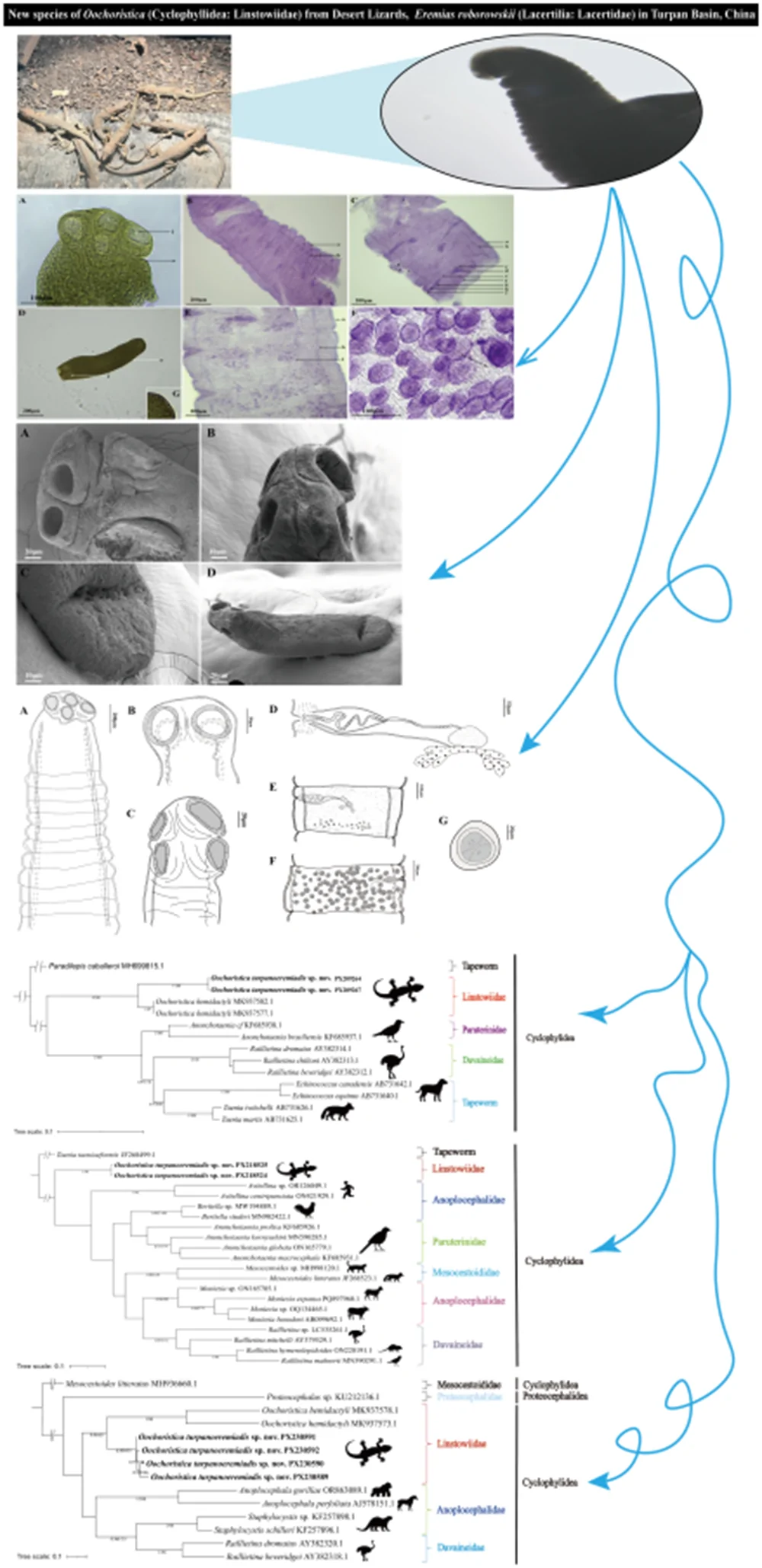

## Introduction

The genus *Oochoristica* Lühe, 1898 belongs to the family Linstowiidae within the order Cyclophyllidea (Cestoda). It is globally distributed and primarily parasitizes reptiles. When Lühe (1898) erected the genus *Oochoristica*, he designated *Oochoristica tuberculata* (Rudolphi, 1819) Lühe, 1898 as the type species; the genus has since undergone considerable expansion. Seventy-five species of the genus *Oochoristica* have been described in reptiles worldwide: 5 from the Australian Realm, 9 from the Ethiopian Region, 14 from the Nearctic Region, 6 from the Neotropical Region, 24 from the Oriental Region and 17 from the Palearctic Bursey and Goldberg (1996). Region. Subsequently, McAllister and Bursey (2017) listed nearly 100 valid species within the genus and delineated their principal diagnostic characteristics. However, there are no detailed records of *Oochoristica* infecting desert lizards in the Palearctic Region of China. This study represents the first record of this genus among desert lizards in China.

This genus presents some problems for the identification of its species, mainly due to the ambiguous morphological diagnostic characteristics, strong phenotypic plasticity of the genus and the lack of sufficient supporting molecular data (Velázquez-Brito et al., 2025). Conventional identification approaches rely heavily on a limited set of morphological traits of mature proglottids, such as the shape of mature segments and testicular count; yet these features are prone to observational errors and exhibit significant variability in response to natural intraspecific variation and environmental factors. For example, the morphological trait pertaining to the shape of mature proglottids in *Oochoristica guerreroensis* Velázquez-Brito, García-Prieto, de Oca et León-Règagnon, 2025 is shared by at least 13 congeneric species (Mašová et al., 2012; Mariaux and Beveridge, 2020). In addition, the testicular number of *O. javaensis* Kennedy, Killick et Beverley-Burton, 1982, exhibits a wide range of 17–46 among different individuals, relying solely on this characteristic to differentiate it from closely related species (Criscione and Font, 2001a). Furthermore, the original morphological descriptions of most species are incomplete, posing great difficulties for subsequent taxonomic comparison; some reported taxa even lack formal nomenclature, which has further exacerbated the taxonomic confusion surrounding this genus (McAllister and Bursey, 2017). Meanwhile, the genus has long been devoid of robust molecular data support, with the vast majority of species lacking available genetic sequences, which has severely hindered the elucidation of its phylogenetic relationships. Although a small number of recent studies (e.g., Verma et al., 2020; Velázquez-Brito et al., 2025) have begun to address this knowledge gap, preliminarily validating the species status of some taxa and revealing a close phylogenetic affinity between *Oochoristica* and *Mathevotaenia* Akumyan, 1946, the genetic information available for taxonomic comparison remains extremely scarce overall. Thus, taxonomic research on this genus is in urgent need of breakthroughs through the integration of morphological data and multi-gene molecular evidence.

*Eremias roborowskii* Bedriaga, 1906 represents a member of *Eremias* Wiegmann, 1834, a genus in the family Lacertidae (Squamata). This species is an endemic desert lizard restricted to the Turpan Basin in Xinjiang, China, and its distribution is closely associated with Gobi shrubland habitats in the southern foothills of the Tianshan Mountains (2024). Dietary studies have demonstrated that *E. roborowskii* is primarily insectivorous, preying predominantly on insects of the orders Coleoptera and Orthoptera, as well as members of Hymenoptera, Acrididae, Carabidae, Tenebrionidae and Formicidae (Wang 2021).

In this study, specimens of the genus *Oochoristica* were recovered from the small intestine of *E. roborowskii*. Through a systematic examination and detailed morphological description, a comparative morphological analysis was conducted against congeneric species distributed worldwide, with a particular focus on elucidating key taxonomic indicators, including the reproductive system and cephalic structures. Using sequences of the nuclear small subunit ribosomal RNA gene (*18S*), *ITS1-5.8S-ITS2* gene cluster and mitochondrial cytochrome *c* oxidase subunit I (*COI*) gene, we inferred phylogenetic relationships. This integrative taxonomic approach was used to clarify the phylogenetic position of the species within the family Linstowiidae, particularly within the genus *Oochoristica*.

## Materials and methods

### Parasite collection

**Figure 1.**
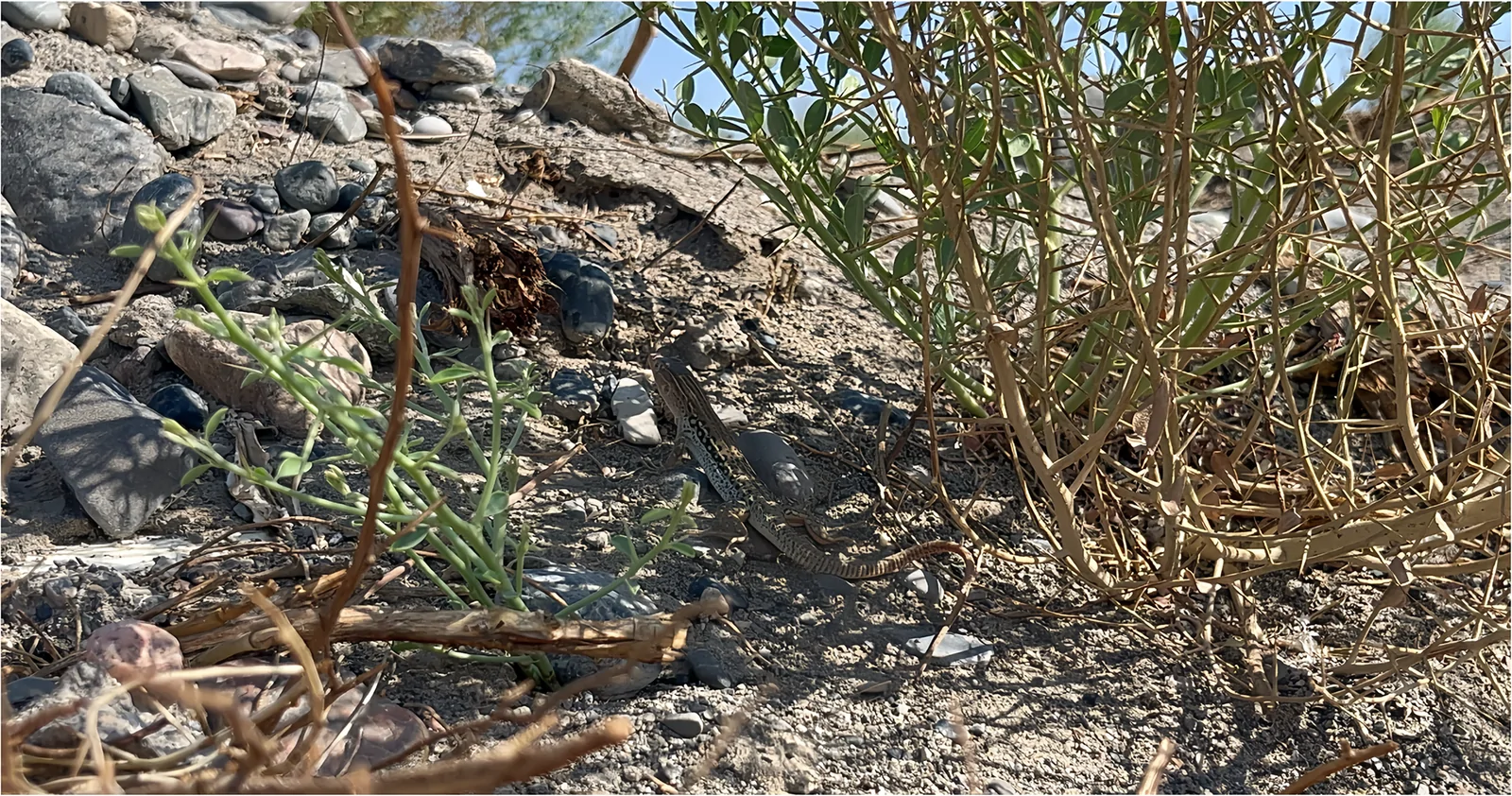
*Eremias roborowskii* and its habitat.

Sixty-seven individuals of *E. roborowskii* were collected from the Turpan Basin, Xinjiang Uygur Autonomous Region, China (Figure 1, Table 1). Of these, 20 specimens were obtained from the herpetological collection of the College of Life Sciences, Xinjiang Agricultural University, where they had been preserved long-term in 4% formalin. The remaining 47 lizards were euthanized via intraperitoneal injection of sodium pentobarbital. Subsequent necropsy revealed adult and larval tapeworms in the small intestine, which were collected and preserved in a glycerol-ethanol solution (5% glycerol, 80% ethanol). The holotype and paratypes were deposited in the National Parasite Resource Center, China, in accordance with international standards for biological specimen preservation, and have been assigned the official accession number (Registration ID: 24F0001ObzS).

**Table 1.** Collection areas and sample size of *Eremias roborowskii* Table 1 long description.

		Sample size (units)		
Sampling site		Sub-adult	Female	Male
Turpan Basin	Gaochang District 89°19’E, 42°85’N	2	9	11
	Shanshan County 88°45’E, 42°85’N	0	8	11
	Toksun County 90°36’E, 42°86’N	3	13	10
Total		5	30	32

### Morphological observation

Following precooling at 4°C, the cestode specimens were heat-fixed in boiling water at 90°C. Mature and gravid proglottids of the cestodes were subjected to staining and microscopic observation via the hematoxylin staining method, with immersion in hematoxylin staining solution overnight. They were then dehydrated through a graded alcohol series (30%, 50%, 60% and 70%), differentiated in 2% acid-alcohol, cleared in xylene and mounted in neutral balsam to prepare permanent slides. Morphological analyses were performed using a digital microscope equipped with an image acquisition and analysis system (Motic BA210). For scanning electron microscopy (SEM), scoleces and larvae were examined using a Zeiss Supra55 VP microscope. Specimens were rinsed in 0.2 mol/L phosphate buffer (PB), fixed in FAA (formaldehyde-acetic acid-ethanol fixative) and rinsed again in PB. After graded ethanol dehydration (50–100%), the samples were freeze-dried and sputter-coated with gold. Observations and micrographs were conducted at an accelerating voltage of 2 kV. Based on the observations from both light microscopy and SEM, line drawings were prepared using Adobe Illustrator 2024 software (Wang et al., 2025; Yan et al., 2025).

### Molecular analysis

In the present study, a total of 5 adult proglottids and 4 cysticercoids were selected for genomic DNA extraction. Genomic DNA of the tapeworm specimens was extracted using a Micro-Sample Genomic DNA Extraction Kit (Tiangen Biotech, Beijing, China) following the manufacturer’s protocols. Three molecular markers were selected for polymerase chain reaction (PCR) amplification: the nuclear small subunit ribosomal RNA gene (*18S*), the *ITS1-5.8S-ITS2* gene region and the mitochondrial cytochrome *COI* gene (Table 2). After quality assessment of the PCR products via electrophoresis on a 1.5% agarose gel stained with GoldView™, the amplicons were sent to Sangon Biotech (Shanghai) Co., Ltd. for purification and bidirectional sequencing using an ABI 3730 DNA sequencer. Raw sequences were assembled and manually edited using DNAMAN 9.0 software. Homology searches were performed via BLAST analysis against the National Center for Biotechnology Information (NCBI) database (https://blast.ncbi.nlm.nih.gov) (Wang et al., 2025). The *18S, ITS1-5.8S-ITS2* and *COI* sequences generated in this study have been deposited in the GenBank database (http://www.ncbi.nlm.nih.gov) with assigned accession numbers.

**Table 2.** Detailed information regarding the primers, reaction systems and reaction conditions for the different target regions of the PCR-amplified molecular markers Table 2 long description.

Region	Name	Sequence (5′→3′)	PCR reaction system	PCR reaction conditions	References
*18S*	Nem*-18S-*F	CGCGAATRGCTCATTACAACAGC	2 μL of DNA template, 1 μL of each of primers, 12.5 μL of 2 × Taq PCR Master Mix II and 8.5 μL of sterilized ultrapure water	An initial denaturation at 95°C for 3 min, followed by 35 cycles of denaturation at 95°C for 30 s, annealing at 52–55°C for 45 s, extension at 72°C for 45 s and a final extension at 72°C for 10 min.	Floyd et al., 2005
	Nem*-18S-*R	GGGCGGTATCTGATCGCC			
*COI*	*COI*-Forward	TTTTTTGGGCATCCTGAGGTTTAT		An initial denaturation at 94°C for 3 min, followed by 35 cycles of denaturation at 94°C for 30 s, annealing at 46–49°C for 30 s, extension at 72°C for 1 min and a final extension at 72°C for 5 min.	Li et al., 2008
	*COI*-Reverse	TAAAGAAAGAACATAATGAAAATG			
*ITS1*–*5.8S-ITS2*	*ITS1-5.8S-ITS2-*F	AGGAATTCCTGGTAAGTGCAAG	3 μL of DNA template, 1 μL of each of primers, 7.5 μL of 2 × Taq PCR Master Mix II and 12.5 μL of sterilized ultrapure water	An initial denaturation at 94°C for 3 min, followed by 35 cycles of denaturation at 94°C for 40 s, annealing at 61–62°C for 30 s, extension at 72°C for 1 min and a final extension at 72°C for 7 min.	Verma et al., 2020
	*ITS1-5.8S-ITS2-*R	CGTTACTGAGGGAATCCTGGT			

### Phylogenetic study

PhyloSuite v1.2.2 software was employed to construct Bayesian Inference (BI) and Maximum Likelihood (ML) phylogenetic trees for the *18S* rRNA, *ITS1-5.8S-ITS2* rRNA and mitochondrial *COI* gene sequences, respectively (Zhang et al., 2021). ML analysis was performed using IQ-TREE, with branch support values evaluated via 1,000 replicates of Ultrafast Bootstrap. In the BI trees, Bayesian Posterior Probability (BPP) ≥0.98 was considered strong support, and 0.95 ≤ BPP < 0.98 was regarded as moderate support; in the ML trees, bootstrap support (BS) ≥80% indicated strong support, while 50% ≤ BS < 80% represented moderate support (Chen et al., 2023). Topological visualization and annotation optimization of all phylogenetic trees were completed in iTOL v7 (Interactive Tree Of Life). Genetic divergence analyses were conducted using MEGA 11.0, and both intraspecific and interspecific genetic distances were calculated based on the uncorrected p-distance model (derived from the proportion of nucleotide differences). The trees were visualized and edited in the iTOL v7 (Interactive Tree of Life). We used *Paradilepis caballeroi* Rysavy and Macko, 1971 (accession numbers: MH699815), *Mesocestoides litteratus* Batsch, 1786 (accession numbers: MH936660) and *Taenia taeniaeformis* Batsch, 1786 (accession numbers: JF268499) as out-groups. Detailed information on the sequences of the genus *Oochoristica* included in the phylogenetic analysis is presented in Tables 3, 4 and 5.

**Table 3.** Cestode species used in the phylogenetic analysis of the *18S* rRNA gene regions of the representatives of the genus *Oochoristica* Table 3 long description.

			GenBank ID	
Species	Host	Distribution	*18S*	References
*Oochoristica turpanoeremiadis* sp. nov.	*Eremias roborowskii*	China	PX209264, PX209267	This study
*Echinococcus canadensis*	Canoidea	United Kingdom	AB731640	Nakao et al., (2013)
*Echinococcus equinus*	Canoidea	Peru and Kazakhstan	AB731642	Nakao et al., 2013
*Taenia twitchelli*	Musteloidea	Russia	AB731626	Nakao et al., 2013
*Taenia martis*	Guloninae	Croatia	AB731625	Nakao et al., 2013
*Anonchotaenia brasiliensis*	*Thraupis cyanoptera*	Brazil	KF685937	Phillips et al., 2014
*Anonchotaenia cf*	*Ammodramus humeralis*	Brazil	KF685938	Phillips et al., 2014
*Raillietina beveridgei*	*Dromaius novaehollandiae*	Australia	AY382312	Unpublished
*Raillietina chiltoni*	*Dromaius novaehollandiae*	Australia	AY382313	Unpublished
*Raillietina dromaius*	*Dromaius novaehollandiae*	Australia	AY382314	Unpublished
*Oochoristica hemidactyli*	*Hemidactylus brookii*	India	MK937582	Verma et al., 2020
	*Hemidactylus brookii*	India	MK937577	Verma et al., 2020
*Paradilepis caballeroi*	*Phalacrocorax*	Mexico	MH699815	Ortega-Olivares et al., (2019)

**Table 4.** Cestode species used in the phylogenetic analysis of the *COI* gene regions of the representatives of the genus *Oochoristica* Table 4 long description.

			GenBank ID	
Species	Host	Distribution	*COI*	References
*Oochoristica turpanoeremiadis* sp. nov.	*Eremias roborowskii*	China	PX218524, PX218525	This study
*Bertiella studeri*	*Homo sapiens*	Sri Lanka	MN982422	Amarasinghe et al., (2020)
*Bertiella* sp.	*Homo sapiens*	Poland	MW194889	Unpublished
*Avitellina* sp.	–	India	OR126049	Upload directly
*Avitellina centripunctata*	Domestic animals	Iraq	ON421929	Unpublished
*Anonchotaenia prolixa*	Passeriformes	Brazil	KF685926	Phillips et al., (2014)
*Anonchotaenia kornyushini*	Aves	Malaysia	MN590285	Upload directly
*Anonchotaenia globata*	–	Iraq	ON165779	Upload directly
*Anonchotaenia macrocephala*	Passeriformes	Brazil	KF685931	Phillips et al., 2014
*Mesocestoides* sp.	*Felis* and *Canis familiaris*	Europe	MH998120	Unpublished
*Mesocestoides litteratus*	*Vulpes vulpe*s	Slovak Republic	JF268523	Hrčkova et al., (2011)
*Moniezia* sp.	–	Spain	ON165789	Upload directly
*Moniezia expansa*	*Caprinae*	India	PQ897960	Unpublished
*Moniezia* sp.	–	India	OQ134465	Upload directly
*Moniezia benedeni*	–	Japan	AB099692	Upload directly
*Raillietina* sp.	–	Japan	LC535261	Unpublished
*Raillietina mitchelli*	*Dromaius novaehollandiae*	Australia	AY379529	Unpublished
*Raillietina hymenolepidoides*	*Psammomys obesus*	Egypt	ON228191	Upload directly
*Raillietina mahnerti*	Aves	Malaysia	MN590291	Upload directly
*Taenia taeniaeformis*	*Vulpes vulpe*s	Slovak Republic	JF268499	Hrčkova et al., 2011

**Table 5.** Cestode species used in the phylogenetic analysis of the *ITS1-5.8S-ITS2* gene regions of the representatives of the genus *Oochoristica* Table 5 long description.

			GenBank ID	
Species	Host	Distribution	*ITS1-5.8S-ITS2*	References
*Oochoristica turpanoeremiadis* sp. nov.	*Eremias roborowskii*	China	PX230589, PX230590, PX230591, PX230592	This study
*Proteocephalus* sp.	*Todarodes pacificus*	South Korea	KU212136	Upload directly
*Staphylocystis* sp.	*Sorex araneus*	American	KF257898	Tkach et al., (2013)
*Staphylocystis schilleri*	*Sorex araneus*	American	KF257896	Tkach et al., 2013
*Oochoristica hemidactyli*	*Hemidactylus brookii*	India	MK937578	Verma et al., 2020
*Oochoristica hemidactyli*	*Hemidactylus brookii*	India	MK937573	Verma et al., 2020
*Anoplocephala gorillae*	*Gorilla beringei beringei*	Republic of Rwanda	OR863089	Unpublished
*Anoplocephala perfoliata*	*Equus caballus*	Germany and United Kingdom	AJ578151	Drogemuller et al., (2004)
*Raillietina dromaius*	*Dromaius novaehollandiae*	Australia	AY382320	Unpublished
*Raillietina beveridgei*	*Dromaius novaehollandiae*	Australia	AY382318	Unpublished
*Mesocestoides litteratus*	*Felis catus*	Germany	MH936660	Unpublished

### Data analysis

Prevalence and mean intensity of infection were calculated following the method described by Yan et al. (2021). Prevalence (%) = (Number of infected samples/Total number of samples) × 100%; Mean intensity of infection = (Sum of infection intensities in positive samples/Total number of positive samples).

## Results

### Systematics

Class: Cestoda Rudolphi, 1808

Order: Cyclophyllidea van Beneden, 1900

Family: Linstowiidae Mola, 1929

Genus: *Oochoristica* Lühe, 1898

Species: *Oochoristica turpanoeremiadis* sp. nov.

### Taxonomic summary

**Type host:** *Eremias roborowskii* Bedriaga, 1906 (Squamata: Lacertidae).

**Type locality:** Turpan Basin, Xinjiang, China.

**Site of infection:** Small intestine.

**Prevalence:** 2.9% (2/67). (2 infected hosts out of 67 analysed).

**Range of infections:** 1–64 parasites per host.

**Mean intensity:** 32.5 parasites per host.

**Representative DNA sequences:** Representative genetic data were deposited in the National Center for Biotechnology Information (NCBl) database (http://www.ncbi.nlm.nih.gov) under the accession numbers: PX209264, PX209267(*18S*); PX218524, PX218525(*COI*); PX230589, PX230590, PX230591 and PX230592 (*ITS1-5.8S-ITS2*).

**ZooBank registration:** The Life Science Identifier for *Oochoristica turpanoeremiadis* sp. nov. is urn:lsid:zoobank.org: pub: 11975BF6-CC5F-461A-BE4D-0AE164E03903.

**Etymology:** The new species is named after the type locality and the generic name of its host species.

### Morphological description

**Figure 2.**
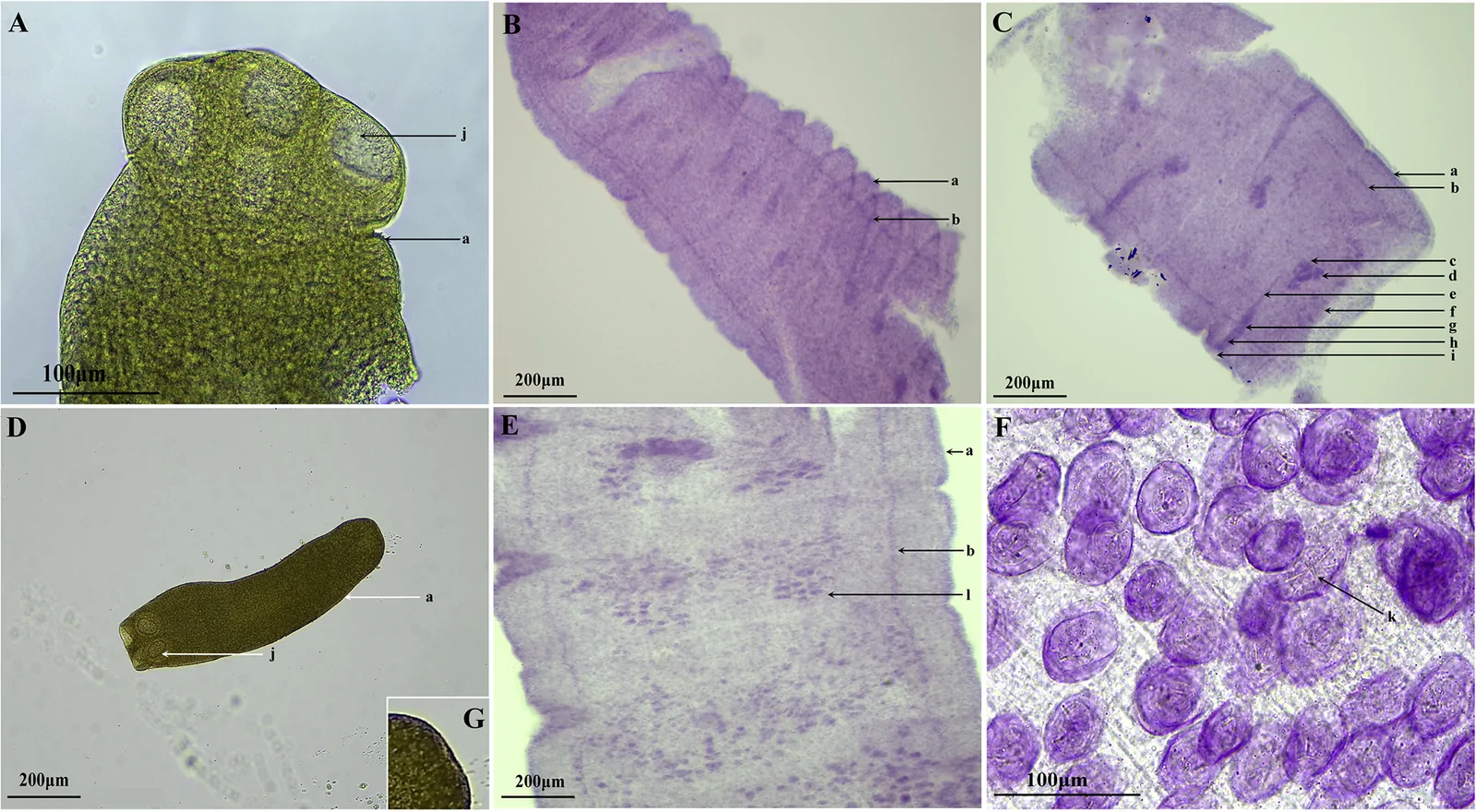
Light micrographs of *Oochoristica turpanoeremiadis* sp. nov. (A) Scolex. (B) Immature proglottid. (C) Mature proglottid. (D) Cysticercus. (E) Gravid proglottid. (F) Egg. (G) Terminal median excretory pore. (a) Tegument. (b) Longitudinal excretory canals. (c) Vitellarium. (d) Ovary. (e) Vagina. (f) Testes. (g) Seminal receptacle. (h) Cirrus. (i) Genital pore. (j) Sucker. (k) Oncosphere hooks.

**Figure 3.**
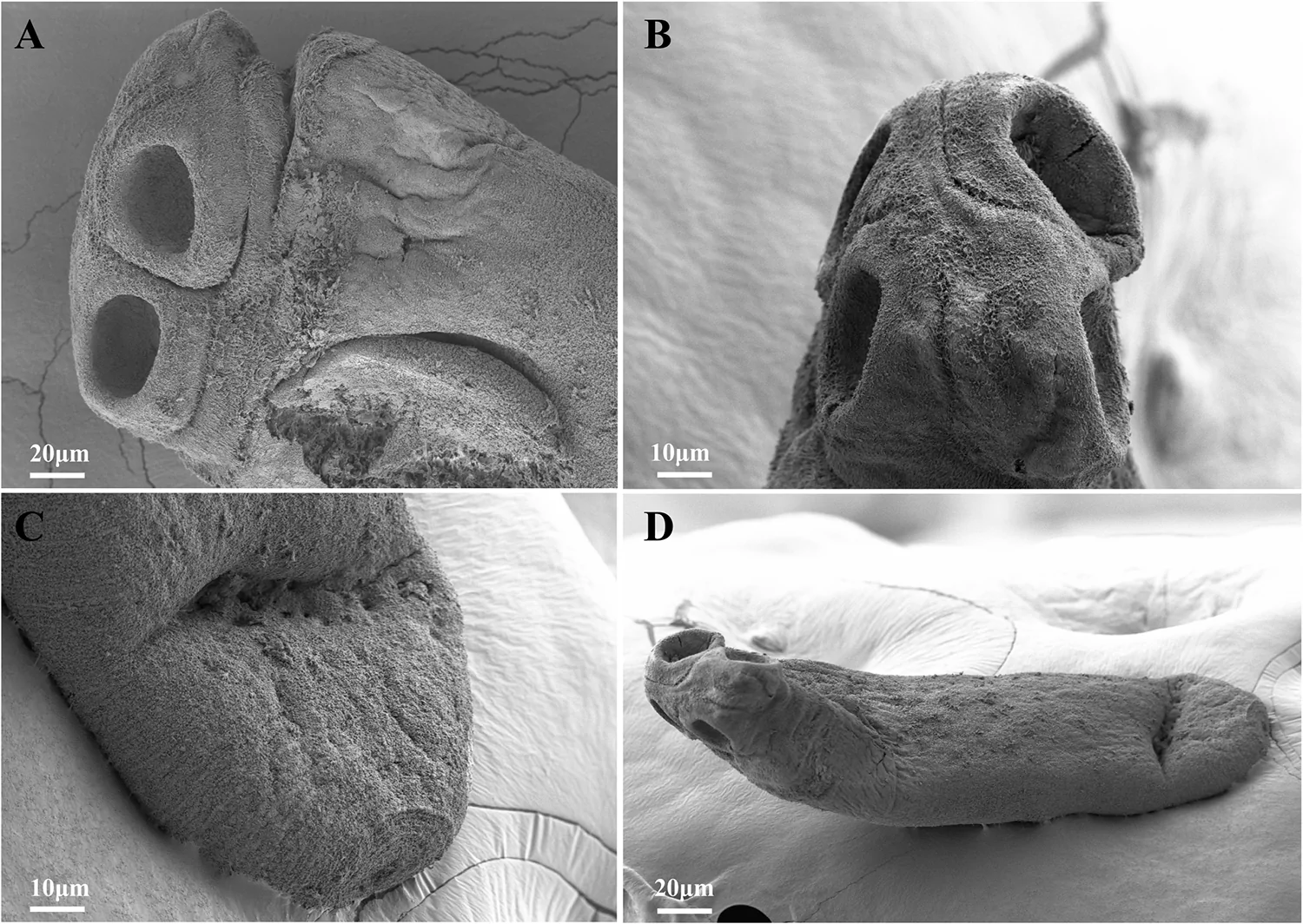
Scanning electron micrographs of the cysticercoid of *Oochoristica turpanoeremiadis* sp. nov. (A) Lateral view of the scolex. (B) Apical view of the scolex. (C) Terminal excretory pore. (D) Cysticercoid.

**Figure 4.**
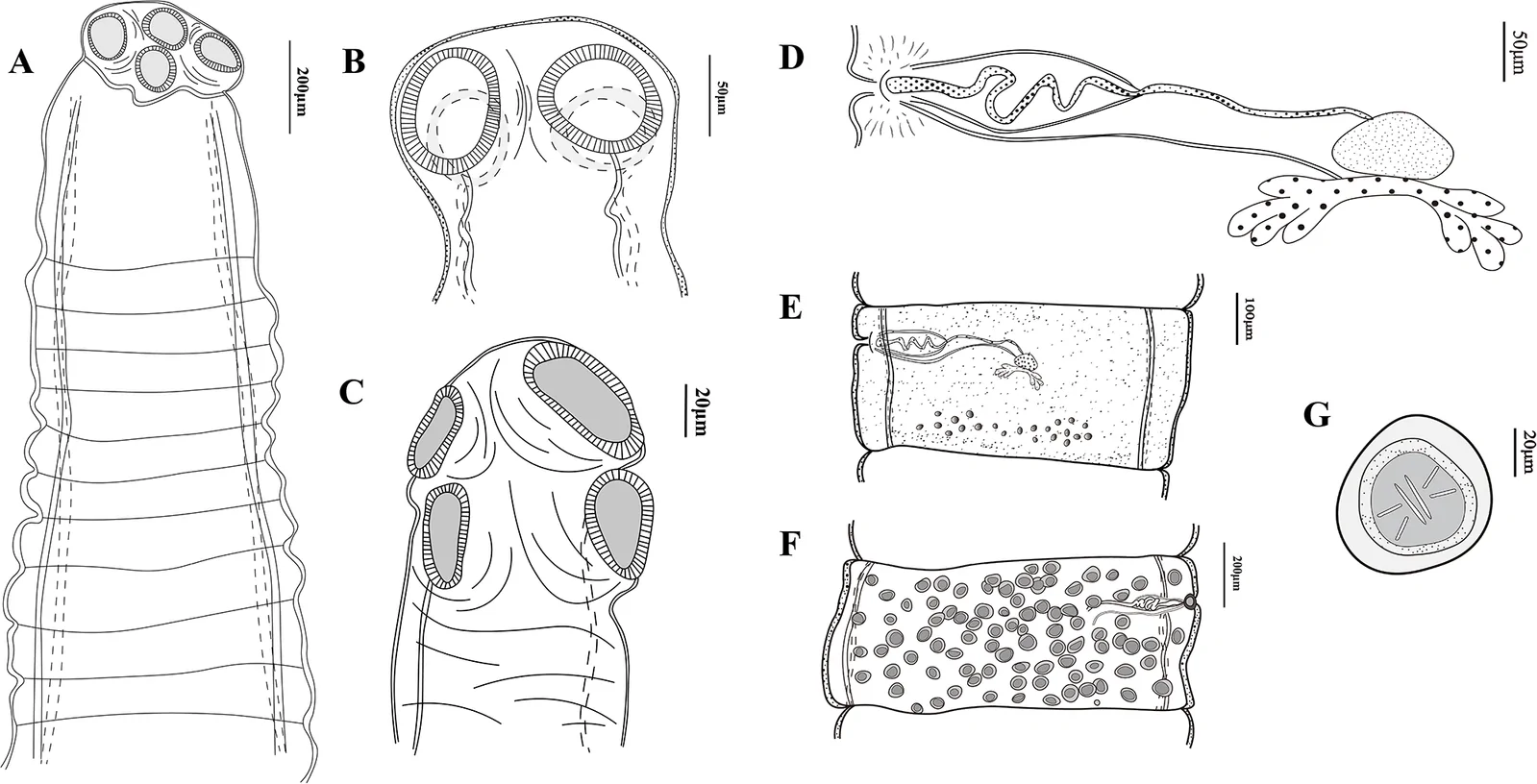
Line Drawings of *Oochoristica turpanoeremiadis* sp. nov.(A) Scolex, neck region and immature proglottids. (B) Scolex suckers. (C) Scolex suckers, apical view. (D) Male and female reproductive organs. (E) Mature proglottid. (F) Gravid proglottid. (G) Egg containing an oncosphere.

The worm is milky white, dorsoventrally flattened and composed of 74 proglottids with a total length of 19.6 mm. The scolex is approximately elliptical, measuring 249.9 ± 28.5 (217.6–283.2) μm in length and 131.3 ± 21.7 (100.9–152.4) μm in width, lacking a rostellum and hooks (Figures 2A, 3B, 4B). Four subelliptical suckers are present, each 79.5 ± 10.7 (74.0–92.8) μm long and 60.9 ± 7.9 (49.0–69.4) μm wide, and the opening of each sucker is slightly concave (Figures 2A, 3A, 4C). A short and slender neck follows the scolex, measuring 0.9 mm in length. Posterior to the neck are the body proglottids, which are short and broad, with the width of each proglottid consistently exceeding its length. A pair of dorsoventral longitudinal excretory canals is present on each side, extending throughout the entire worm (Figure 2B). A total of 19 immature proglottids are present, measuring 88.4 ± 4.9 (82.9–92.2) μm in length and 534.8 ± 68.8 (469.6–606.7) μm in width. The mature proglottids number 22, measuring 217.8 ± 37.2 (186.0–258.6) μm in length and 686.5 ± 12.2 (679.2–700.6) μm in width. Each mature proglottid contains a single set of hermaphroditic reproductive organs. Testes are spherical or ovoid (12.4–28.5 μm in diameter), 20–25 per proglottid, distributed along the posterior margin of the proglottid, posterior to and on both sides of the vitellarium (Figures 2F, 4E). The cirrus is highly convoluted (Figures 2H, 4D). The seminal receptacle is fusiform, measuring 137.7 ± 10.9 (122.9–152.2) μm in length and 29.2 ± 2.6 (25.4–32.4) μm in width (Figures 2G, 4D). Genital pores are located on the lateral margins of the proglottids, alternating irregularly between the left and right sides. The ovary is bilobed, 46.3–90.2 μm in length and 17.1–27.6 μm in width; each primary lobe is further subdivided into 4 to 5 subsidiary lobules. (Figures 2D, 4D). The vitellarium is elliptical, 27.5–53.2 μm in length and 12.6–26.2 μm in width, situated anterior to the ovary (Figures 2C, 4D). The vagina is located posterior to the vas deferens (Figures 2E, 4D). The gravid proglottids number 31, measuring 447.4 ± 137.8 (335.9–601.4) μm in length and 1017.9 ± 56.2 (977.2–1082.0) μm in width (Figures 2E, 4F), are filled with eggs (Figures 2F, 4G). The eggs are spherical with a diameter of 31.8–56.8 μm, encased within a bilayered eggshell that is characterized by a thicker inner lamella. Each egg encloses an oncosphere, the hooks of which measure 12.7 (11.0–14.7) μm in length.

The cysticercoid of this tapeworm is milky white, measuring 762.4 ± 54.1 (676.8–849.7) μm in length and 215.2 ± 24.1 (185.7–273.3) μm in width. The body wall is smooth, densely covered with annular transverse striations and lacks distinct accessory structures; proglottids are undifferentiated (Figure 3D). Four subelliptical suckers are located at the anterior end of the body, with dimensions of 52.06 ± 6.42 (42.2–61.9) μm in length and 40.2 ± 5.3 (31.6–46.2) μm in width, and no rostellum or hooks are present (Figure 3A, B). The posterior end is obtusely rounded, with an excretory pore located at the midline of the terminal body (Figures 2G, 3C). The body cavity contains granular structures (Figure 2D).

### Remarks

The present specimen exhibited the emended diagnostic morphological characteristics typical of the genus *Oochoristica* ((1994); Mašová et al., 2012): lacking interproglottidal glands; a scolex without hooks and armed with 4 suckers; a single genital pore arranged in an irregular alternating pattern; and a large number of testes located posterior to and on both sides of the vitellarium.

Although the new species described herein, *O. turpanoeremiadis* sp. nov., shares some morphological similarities with the type species, *O. tuberculata* (Rudolphi, 1819) Lühe, 1898, its body size is significantly smaller. Furthermore, *O. turpanoeremiadis* exhibited distinct and significant differences in morphological characteristics compared to the 8 closely related congeners (Table 6). The main differences are as follows:

**Table 6.** Comparative morphological characteristics of *Oochoristica* tapeworms Table 6 long description.

Insect species	*O. turpanoeremiadis*	*O. hemidactyli*	*O. beveridgei*	*O. leonregagnonae*	*O. chalcidesi*	*O. harschi*	*O. whitfieldi*
Host	*Eremias roborowskii*	*Hemidactylus brookii Gray*	*Tarentola parvicarinata Joger*	*Ctenosaura pectinata osaura pectinata*	*Chalcides ocellatus*	*Hemidactylus turcicus*	*Ctenosaura oaxacana*
References	This study	Verma et al., 2020	Mašová et al., 2010	Arizmendi-Espinosa et al., 2005	Schuster, 2011	McAllister and Bursey, 2017	Guillén-Hernández et al., 2007
Site of infection	Small intestine	Intestine	Small intestine	Intestine	Small intestine	Small intestine	Intestine
Body length (mm)	19.55	49–53	37	57.6	15–20	65	14.4–33.7
Body width (mm)	0.47–1.08	0.62–0.79	0.595–0.661	–	1	0.54	–
Proglottids	74	23	79	98	59–69	145	29–37
Scolex length	217.59–283.20	–	224–400	–	–	110	250–260
Scolex width	100.88–152.43	152–187	360–420	0.5–0.8	420–480	170	340–370
Sucker length	74.01–92.79	75–81	116–154	190–230	138–200	100	142–165
Sucker width	48.99–69.35	75–81	111–147	150–190	138–195	70	107–132
Neck length (mm)	0.88	0.77	753–945	–	270–700	5.5	750–1,000
Immature proglottids length	82.91–92.23	–	130–178	200–1,250	–	180	100–400
Immature proglottids width	469.56–606.65	–	366–375	700–2,200	–	100	220–430
Mature proglottids length	185.95–258.62	–	668–868	600–2,000	550–810	380	450–1,000
Mature proglottids width	679.19–700.59	–	554–589	1,100–2,020	600–880	380	350–690
Gravid proglottids length	335.88–601.42	–	1,153–1,608	1,200–3,000	1,000–1,050	1,550	1,450–2,250
Gravid proglottids width	977.19–1082.04	–	339–627	1,200–2,300	950–1,000	540	400–710
Testes	20–25	20–30	35–45	78–112	25–35	14–20	28–45
Testes diameter	12.42–28.50	35–60	33–45	35–40	25–35	24	70–107
Seminal receptacle length	122.94–152.17	–	107–183	180–230	150–170	40	110–150
Seminal receptacle width	25.36–32.42 (19.28–27.02)	37–69	67–93	50–70	50–70	20	42–54
Ovary length	46.31–90.22	–	175–197	100–450	–	140	49.4–160.5
Ovary width	17.09–27.64	98–231	127–202	200–600	158–175	70	103.7–180.3
Vitellaria length	27.49–53.18	180–270	53–114	60–300	33–50	73	40–100
Vitellaria width	12.59–26.18	180–270	86–129	160–400	88–100	73	40–90
Egg diameter	31.79–56.83	37–49	–	30–40	63–70	55–75	19–23
Oncosphere diameter	–	25–28	43–47	20–30	35–38	31–43	15–20
Oncosphere hooks length	10.96–14.72 (10.96–14.72)	25–28	15–17	10–20	20	16	10–14

All measurements are presented as ranges, except for those of *O. harschi*, which are given as exact values. Unless otherwise stated, measurements are in micrometres (μm).

*O.turpanoeremiadis* sp. nov. is morphologically the most similar to *O. chalcidesi* Schuster, 2011, a congener from the Palearctic Region of the United Arab Emirates. However, *O. chalcidesi* possesses a subglobular scolex measuring 420–480 μm in width; it has 25–35 testes distributed in the mid-region of the proglottid; the eggs are oval in shape, measuring 63–70 μm in diameter, with the hooks approximately 20 μm long (Schuster, 2011).

*O. turpanoeremiadis* sp. nov. differs markedly from species of the Neotropical Region in both body morphology and testicular number. The new species has a total length of 19.6 mm (74 proglottids), with gravid proglottids wider than long. In contrast, *O. guerreroensis* is considerably shorter (4.5–7.4 mm), has fewer proglottids (31–39) and its gravid proglottids are longer than wide (Velázquez-Brito et al., 2025). Furthermore, there are striking differences in testicular count: *O. leonregagnonae* Arizmendi-Espinosa, Garcia-Prieto et Guillen-Hernandez, 2005 possesses 78–112 testes (Arizmendi-Espinosa et al., 2005), *O. whitfieldi* Guillen-Hernandez, Garcia-Prieto et Arizmendi-Espinosa, 2007 has 28–45 (Guillén-Hernández et al., 2007), and *O. Beveridgei* Masova, Tenora, Barus et Koubek, 2010 has 22–32 (Goldberg et al., 2007). Conversely, the new species described herein has a relatively low number of testes (20–25) per mature proglottid.

In comparison with species from the Ethiopian Region, *O. beveridgei* possesses 35–45 testes, which is more than that of the new species. Furthermore, its gravid proglottids are longer than wide, which is the opposite of the new species (Mašová et al., 2010). From the Nearctic Region: *O. harschi* McAllister and Burseyis, 2017, is shorter (10–15 mm) but has more numerous proglottids (145–150). Its suckers are circular, and it has fewer testes (14–20) arranged in 2 clusters. In contrast, the testes of the new species are arranged in a single cluster confined to the posterior region of the proglottid (McAllister and Bursey, 2017). From the Oriental Region: *O. hemidactyli* Johri, 1955 possesses a rostellum with hooks, and its testes are also arranged in 2 clusters. However, the new species lacks a rostellum and hooks (Verma et al., 2020).

### Phylogenetic analysis

**Figure 5.**
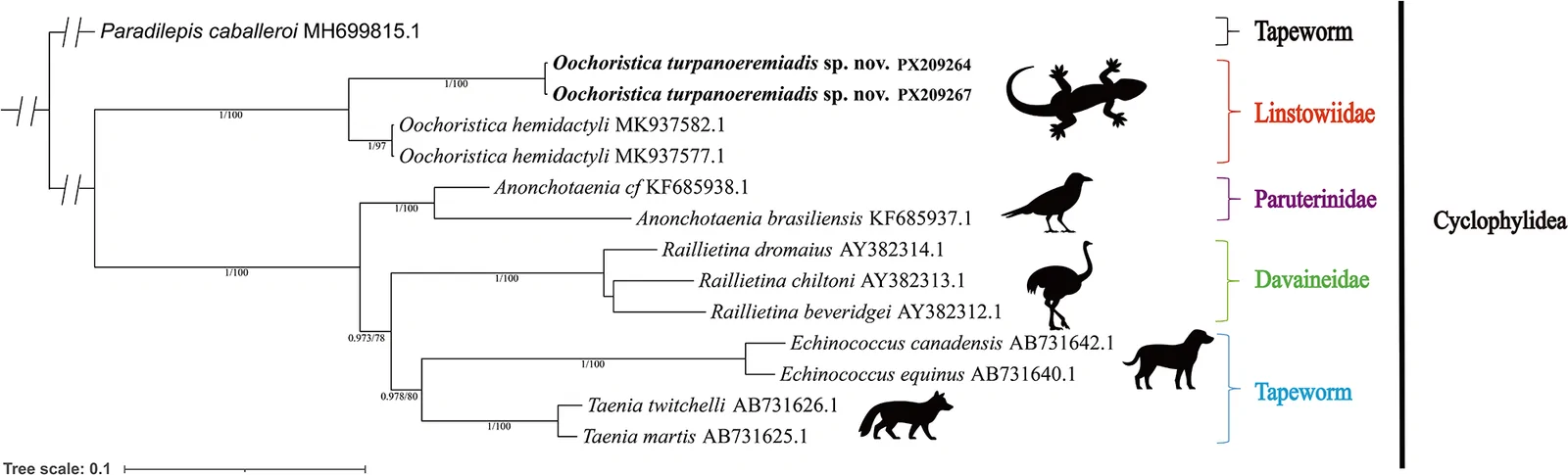
Phylogenetic trees of *O. turpanoeremiadis* sp. nov. from *Eremias roborowskii* (Squamata: Lacertidae), inferred from the concatenated *18S* dataset using BI and ML methods. Node support values are indicated as BPP/BS. PX209264 represents the adult sequence; PX209267 represents the larval sequence. Figure 5 long description.

**Figure 6.**
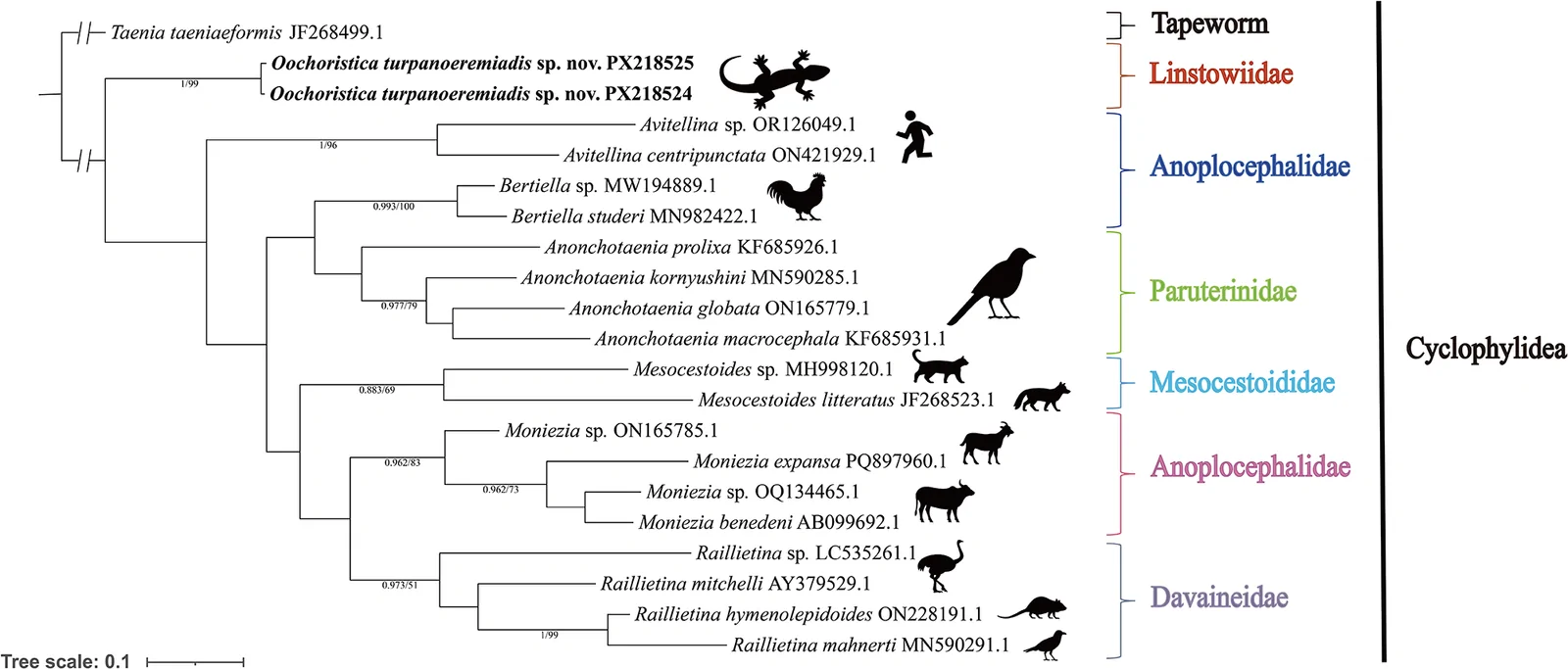
Phylogenetic trees of *O. turpanoeremiadis* sp. nov. from *Eremias roborowskii* (Squamata: Lacertidae), inferred from the concatenated *COI* dataset using BI and ML methods. Node support values are indicated as BPP/BS. PX218525 represents the adult sequence; PX218524 represents the larval sequence. Figure 6 long description.

**Figure 7.**
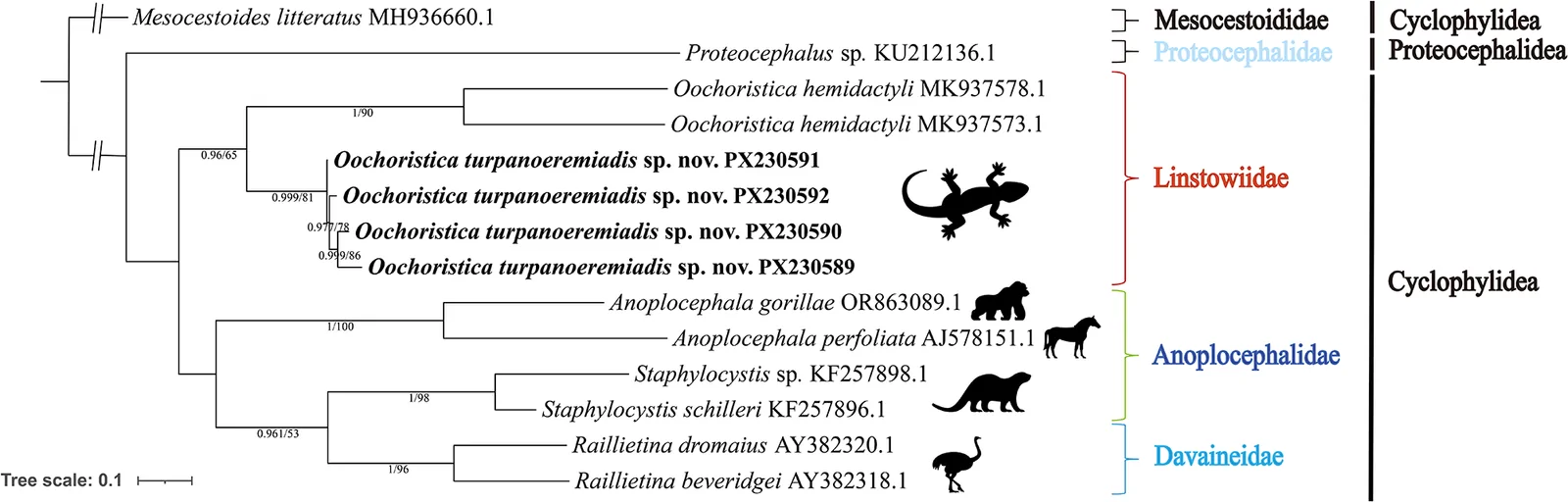
Phylogenetic trees of *O. turpanoeremiadis* sp. nov. from *Eremias roborowskii* (Squamata: Lacertidae), inferred from the concatenated *ITS1-5.8S-ITS2* dataset using BI and ML methods. Node support values are indicated as BPP/BS. PX230591 and PX230592 represent the adult sequence. PX230589 and PX230590 represent the larval sequence. Figure 7 long description.

Phylogenetic trees were constructed using BI and ML methods based on the *18S* rRNA, *ITS1-5.8S-ITS2* and *COI* gene sequences (Figures 5–7). The BI and ML trees inferred from the *18S* dataset were highly congruent in topology. They revealed that adults and cysticercoids of *O. turpanoeremiadis* sp. nov. formed a distinct, monophyletic clade, which was recovered as the sister group to *O. hemidactyli*. This topological relationship received maximum statistical support in both analyses (BPP = 1; BS = 100) and was clearly separated from species of other genera. In the BI and ML trees based on the *COI* gene, intrageneric comparison was not possible due to the paucity of available *COI* sequences for the genus *Oochoristica* in the GenBank database. Nevertheless, the sequences of adult and cysticercoid specimens of *O. turpanoeremiadis* formed an independent clade without intraspecific divergence, clearly distinct from species of other genera, with significant nodal support (BPP = 1; BS = 99). The phylogenetic analyses of the *ITS1-5.8S-ITS2* dataset yielded results consistent with those of the *18S* dataset. *O. turpanoeremiadis* sp. nov. was resolved as the sister taxon to *O. hemidactyli* and formed a distinct lineage separate from other genera. Nevertheless, the nodal support values were relatively lower than those obtained from the *18S* dataset (BPP = 0.96; BS = 81). *18S* is a highly conserved nuclear gene with a slow evolutionary rate and low sequence variation, resulting in stable phylogenetic signals and the highest node support. *COI* as a mitochondrial gene evolves at a moderate rate and exhibits strong performance in species delimitation. In contrast, the *ITS* region is a rapidly evolving nuclear intergenic spacer with high variation within and among closely related species, which leads to decreased node support in some parts of the tree. Collectively, the multi-locus phylogenetic results demonstrate the distinct phylogenetic independence and taxonomic validity of *O. turpanoeremiadis* sp. nov.

### Genetic distances

O. turpanoeremiadis sp. nov. exhibits significant genetic differentiation from congeneric and closely related species based on both ribosomal and mitochondrial sequences (Supplementary Tables 1–3). Analysis of the *18S* rRNA gene sequences revealed an intraspecific genetic distance of 0.0090 among individuals of the new species, whereas the interspecific distance between O. turpanoeremiadis and O. hemidactyli ranged from 0.0671 to 0.0678, supporting its status as an independent evolutionary unit. The *ITS* sequences further corroborated this conclusion. The intraspecific genetic distances within O. turpanoeremiadis were extremely low, ranging only from 0.0035 to 0.0104. In stark contrast, the interspecific distances between O. turpanoeremiadis and O. hemidactyli – a species with similar morphological characteristics – reached 0.2742–0.2756, indicating substantial differentiation at the nuclear gene level. As a mitochondrial marker, the *COI* gene also demonstrated large genetic distances between O. turpanoeremiadis and other genera within the same family (e.g., Beriella, Aviiellina, Mesocestoides), ranging from 0.1435 to 0.2243. These considerable genetic differences further support the distinct phylogenetic independence of O. turpanoeremiadis sp. nov.

## Discussion

Morphological comparisons revealed that the cestode species investigated in the present study differs from all previously described congeners within the genus *Oochoristica* in the length-to-width ratio of the proglottids, the number and distribution of testes, the relative position of the vitelline gland to the ovary, as well as the presence or absence of a rostellum on the scolex. Meanwhile, morphological traits, including the absence of an apical rostellum and small hooks, and the possession of oval suckers in its cysticercoid stage, are consistent with those of the adult individuals. The morphological identification of species within the genus *Oochoristica* is confronted with multifaceted challenges, which severely compromise the accuracy of species delineation and the stability of its taxonomic system, which are mainly manifested in the following aspects:

First, morphological characteristics are highly susceptible to various confounding factors. Differences in the fixation and preservation methods can lead to varying degrees of shrinkage. For instance, specimens of *O. javaensis* fixed *in situ* exhibit severe shrinkage, resulting in significant discrepancies in proglottid dimensions and organ proportions compared with normally fixed specimens (Criscione and Font, 2001a). Criscione and Font (2001a, 2001b) demonstrated that relying solely on testicular number as a diagnostic characteristic might be unreliable, as this trait can fluctuate widely (17–46) in *O. javaensis*. Additionally, scolex size and developmental stage can be influenced by crowding. By comparison, the present taxonomic investigation of *O. turpanoeremiadis* revealed a stable testicular number of 20–25. This narrow numerical range presents negligible intraspecific variation and is barely affected by environmental fluctuations and specimen fixation protocols, thus representing a robust and definitive diagnostic trait for species identification. Brooks and Mayes (1976) confirmed that the body size of tapeworms was inversely proportional to the number of worms infecting the host. Second, morphological criteria for species delimitation are often ambiguous. Core features such as testicular number and sucker shape exhibit extensive overlap among species. For example, *O. harschi* and *O. macallisteri* Bursey and Goldberg, 1996 have similar ranges of testicular numbers and can only be differentiated by auxiliary features such as the presence or absence of a neck and sucker shape (McAllister and Bursey, 2017). Furthermore, many early species descriptions lack critical details regarding the reproductive system and larval characteristics, making it difficult to distinguish between closely related taxa. This has led to misidentification and cases of synonymy, such as *O. gallica* Dollfus, 1954, which was erroneously considered a synonym of *O. tuberculata* (Della Santa, 1956). In contrast, the morphological traits of adults and cysticercoids investigated in the present study are morphologically conservative and highly consistent, which effectively remedies the deficiency of inadequate larval morphological information in conventional taxonomic systems. Furthermore, historical limitations are particularly salient. These include poor specimen preservation and the reliance on hand-drawn illustrations rather than photomicrographic evidence. For example, Beddard’s (1914) original description of *O. ameivae* (Beddard, 1914) Baer, 1924 was compromised by specimen shrinkage, and the hand-drawn figures failed to accurately reflect fine morphological structures. Inconsistent identification criteria among researchers have also hindered effective morphological comparisons across geographical regions and hosts. This is particularly pronounced in closely related groups; for instance, *Oochoristica* and *Mathevotaenia* are morphologically highly similar, and traditional differentiation relied on a limited number of features, such as proglottid overlap, further increasing the difficulty of accurate identification (Spasskii, 1951; Dollfus, 1954; Beveridge, 1994). Based on the definitive morphological indicators identified for the species in the present study, including a testicular number of 20–25, vitelline glands positioned anterior to the ovary, an unarmed scolex lacking a rostellum and hooks, and proglottids distinctly broader than long, these traits can serve as reliable reference criteria to resolve the morphological identification difficulties within the genus *Oochoristica* and improve the accuracy of species discrimination.

Molecular sequencing represents a potentially effective approach for the species identification of the genus *Oochoristica*. However, the availability of gene sequences for *Oochoristica* species in the GenBank database remains extremely scarce to date, highlighting an urgent need to enrich and expand the molecular data for this genus.

The phylogenetic evolution of *Oochoristica* is closely associated with that of its hosts. In terms of the phylogenetic relationships among the hosts, although *O. turpanoeremiadis* and its sister species *O. hemidactyli* parasitize hosts from different families (the lacertid *E. roborowskii* and the gekkonid *Hemidactylus brookii* [Verma et al., 2020], respectively), both hosts belong to the suborder Lacertilia. The phylogenetic relatedness of the hosts provides a basis for the genetic homology of the parasites. This phylogenetic relatedness among host groups provides a foundation for the genetic homology of the parasites, analogous to the close relationship between *O. beveridgei* and *O. junkea* Johri, 1950, both parasitizing gekkonid hosts, as reported by Mašová et al. (2010). Regarding host geographic distribution: *E. roborowskii* is an endemic species restricted to the desert regions of the Turpan Basin in Xinjiang, China (Palearctic Realm). Despite the large geographical distance separating it from *H. brookii* (the host of *O. hemidactyli*, distributed in Meerut, India), the parasites formed a sister group relationship. This finding is consistent with the transcontinental relationship observed between the African *O. beveridgei* and the Asian *O. junkea* (Mašová et al., 2010), reflecting the crucial influence of host geographic dispersal on parasite evolution. Furthermore, the stable distribution range of *E. roborowskii* likely restricts gene flow in *O. turpanoeremiadis*, thereby maintaining genetic independence.

The discovery of *O. turpanoeremiadis* sp. nov. not only elucidates the evolutionary and divergent trajectory of *Oochoristica* in the Palearctic Realm but also deepens the understanding of the association between the geographic isolation mechanism and speciation in the arid regions of Central Asia. As a major distribution area of the genus *Oochoristica*, the Palearctic Realm harbors most extant species in relatively humid or Mediterranean climatic zones, such as Western Asia (*O. chalcidesi* from the United Arab Emirates) and Europe (*O. tuberculata* from Turkey and *O. gallica* from France). In comparison, the species described herein inhabits the Turpan Basin, a long-term geographically isolated area surrounded by the Tianshan Mountains and the Taklamakan Desert. Remarkable genetic divergence exists between the present species and *O. chalcidesi* from West Asia, which is collectively driven by geographic isolation and ecological specialization. On the one hand, the vast deserts and mountain ranges across the Central Asian arid zone block gene exchange among congeneric species, facilitating the independent evolution of populations in the Turpan Basin. This result is consistent with the phenomenon proposed by Hui-Yuan et al that non-native parasites are unable to colonize arid areas, demonstrating that geographic barriers in arid Central Asia impose long-term constraints on parasite dispersal (Hui-Yuan et al., 2016). On the other hand, the adaptation of *E. roborowskii* in Turpan to extremely arid habitats further promotes the interspecific differentiation of these cestodes.

As the first documented case of *Oochoristica* cestode infection in the Turpan Basin of China, this discovery makes an irreplaceable and distinctive contribution to understanding parasitic diversity in the arid regions of Central Asia. This unique desert ecosystem harbors a rich assemblage of endemic flora and fauna, yet research on its parasitic diversity has long remained in a lagging state – prior to this study, only infections by *Dibothriocephalus* spp. (diphyllobothriid tapeworms) had been recorded in the Shardara Reservoir of Kazakhstan, with such investigations focusing exclusively on aquatic ecosystems (Baltabay et al., 2025). Additionally, there have been reports of infections by the nematodes *Abbreviata* spp. (Tao, 1985; Yan et al., 2025) and *Parapharyngodon* spp. (Wang et al., 2025). In this region, the faunal composition and evolutionary history of reptile-associated parasites have not yet been subjected to systematic elucidation. As a core area in the eastern part of the Central Asian arid zone, the Turpan Basin serves as a pivotal biogeographic corridor linking the East Asian and Central Asian biotas. The discovery of this new species fills the distribution gap of the genus *Oochoristica* in this region and corroborates that the Central Asian arid zone is not a barren zone for parasitic diversity, but rather harbours a distinctive assemblage of endemic taxa. This conclusion, together with the archaeological evidence indicating that Silk Road travellers once introduced non-native parasites into the Central Asian arid zone, yet only locally adapted species were able to establish viable populations, forms an integrated historical-modern research framework for parasitic diversity in the Central Asian arid regions (Hui-Yuan et al., 2016).

## Conclusions

A new tapeworm species, *O. turpanoeremiadis* sp. nov., was recovered from the small intestine of *E. roborowskii*, an endemic reptile of the Turpan Basin, Xinjiang, China. Species delimitation was supported by an integrative taxonomic approach combining comparative morphology, molecular phylogenetics and geographical distribution, thereby increasing the robustness of the identification. In the future, more extensive sampling and comparative studies should be conducted on this genus, with particular attention paid to differences in the ultrastructure of the reproductive system, life cycle characteristics, and ecological adaptability, to further improve the classification system of *Oochoristica*.

## Supporting information

10.1017/S0031182026102315.sm001 Yan et al. supplementary material 1 Yan et al. supplementary material

10.1017/S0031182026102315.sm002 Yan et al. supplementary material 2 Yan et al. supplementary material

10.1017/S0031182026102315.sm003 Yan et al. supplementary material 3 Yan et al. supplementary material

## Data Availability

The sequence data generated in this study have been submitted to the GenBank database under accession numbers PX209264, PX209267, PX218524, PX218525 and PX230589-PX230592. The data that support the findings of this study are available from the corresponding author upon reasonable request.

## Supplementary material

The supplementary material for this article can be found at https://doi.org/10.1017/S0031182026102315.

## Figure 5 Long description

The image is a phylogenetic tree illustrating evolutionary relationships within the Cyclophyllidea order. At the top, Paroniopsis catenifer MH899815.1 serves as the outgroup. Below, Oochoristica turpanoeremiadis sp. nov. PX209264 and PX209267 form a distinct clade, indicating a new species closely related to Oochoristica hemidactyli and Oochoristica beijingensis. The tree branches further to show relationships among other species, including Anomotaenia and Raillietina species, as well as Echinococcus and Taenia species. The tree is divided into sections by colored lines representing different families: Linstowiidae, Paruterinidae, Davaineidae and Taeniidae. Animal icons, such as a lizard, bird, ostrich and cat, are used to represent host groups associated with these families. Branch support values are indicated on the tree, providing statistical confidence for the relationships shown.

Navigate back to Figure 5.

## Figure 6 Long description

The phylogenetic tree illustrates the evolutionary relationships among various species within the order Cyclophyllidea, specifically focusing on tapeworms. The tree is oriented vertically, with branches extending from top to bottom. At the top, the tree begins with ′Taenia asiatica/cf. formosana′ as the outgroup. Below this, ′Oochoristica turpanoeremiadis sp. nov. PX218525′ and ′Oochoristica turpanoeremiadis sp. nov. PX218524′ form a distinct clade. The tree further branches into several families, each represented by different species. The Linstowiidae family includes ′Arostrilepis sp. ON248902.1′ and ′Bertiella sp. MW149584.1′. The Anoplocephalidae family is represented by ′Anoplocephaloides cf. variabilis ON248901.1′ and ′Anoplocephaloides kontrimavichusi KF562520.1′. The Paruterinidae family includes ′Anoplocephaloides sp. ON248903.1′. The Mesocestoididae family is represented by ′Mesocestoides sp. ON716545.1′. The Anoplocephalidae family reappears with ′Moniezia expansa KF562521.1′ and ′Moniezia benedeni KF562522.1′. The Davainidae family includes ′Raillietina tetragona ON248904.1′ and ′Raillietina echinobothrida ON248905.1′. Each branch is labeled with the species name and accession number and silhouettes of various animals are depicted alongside the branches to indicate the host species. The tree includes a scale bar representing a time scale of 0.1. Different colors are used to denote the various families: Linstowiidae in red, Anoplocephalidae in blue, Paruterinidae in green, Mesocestoididae in purple, Anoplocephalidae in pink and Davainidae in orange.

Navigate back to Figure 6.

## Figure 7 Long description

The phylogenetic tree is oriented vertically, displaying evolutionary relationships among species within the Cyclophyllidea order. At the top, Mesocestoides litteratus is the outgroup. The tree branches into several clades, with Protocestoides sp. and Oochoristica hemidactyli forming a distinct group. Oochoristica turpanoeremiadis sp. nov. is represented by multiple sequences (PX230591, PX230592, PX230589, PX230590) and forms a monophyletic clade, indicated by silhouettes of lizards. This clade is part of the Linstowiidae family, marked in red. Below, Anoplocephala perfoliata and Anoplocephala gorillae are grouped under Anoplocephalidae, marked in green. Staphylocystis furcata and Raillietina species are part of the Davaineidae family, marked in blue. The tree includes support values at nodes and a scale bar at the bottom indicates a genetic distance of 0.1.

Navigate back to Figure 7.

## Table 1 Long description

The table reports counts of Eremias roborowskii individuals sampled at three locations in the Turpan Basin, grouped by life stage and sex (subadult, female, male). Overall totals are 5 subadults, 30 females, and 32 males, indicating males are slightly more numerous than females and subadults are rare. Gaochang District has 2 subadults, 9 females, and 11 males. Shanshan County has no subadults, with 8 females and 11 males. Toksun County has the highest female count at 13 and the highest subadult count at 3, with 10 males. Male counts are similar across sites, while female counts vary more, and subadults appear only in Gaochang and Toksun. Coordinates are provided for each site, but the table does not describe sampling effort or timing, so differences may reflect collection conditions rather than population structure.

Navigate back to Table 1.

## Table 2 Long description

The table lists PCR primer pairs, their nucleotide sequences, reaction mix components, cycling conditions, and literature sources for three molecular marker regions: 18S, COI, and ITS1 to 5.8S to ITS2. For 18S, primers Nem-18S-F and Nem-18S-R use a reaction mix with 2 microliters DNA template, 1 microliter of each primer, 12.5 microliters of 2× Taq PCR Master Mix II, and 8.5 microliters water, with annealing between 52 and 55 degrees Celsius and a final extension of 10 minutes, cited to Floyd and colleagues, 2005. For COI, primers COI-Forward and COI-Reverse use cycling with annealing between 46 and 49 degrees Celsius and a final extension of 5 minutes, cited to Li and colleagues, 2008; the reaction mix is not specified in the table. For ITS1 to 5.8S to ITS2, primers ITS1 to 5.8S to ITS2-F and ITS1 to 5.8S to ITS2-R use 3 microliters DNA template, 1 microliter of each primer, 7.5 microliters of 2× Taq PCR Master Mix II, and 12.5 microliters water, with annealing between 61 and 62 degrees Celsius and a final extension of 7 minutes, cited to Verma and colleagues, 2020. Across protocols, all use an initial denaturation step followed by 35 cycles and an extension step at 72 degrees Celsius, but annealing temperatures and final extension times differ by marker. Interpretation note: some fields are incomplete for COI, so direct comparison of reaction mixture volumes across all regions is limited.

Navigate back to Table 2.

## Table 3 Long description

The table lists cestode taxa included in an 18S rRNA phylogenetic dataset, giving each species’ host, geographic distribution, GenBank accession ID, and literature source. It includes Oochoristica turpanoeremiadis sp. nov. from the lizard Eremias roborowskii in China, with two accessions (PX209264 and PX209267) reported in the current study. Other entries span multiple genera, including Echinococcus (canids; United Kingdom, Peru, and Kazakhstan) and Taenia (mustelids; Russia and Croatia), all sourced to Nakao and colleagues, 2013. Several bird-associated taxa are included, such as Anonchotaenia from Brazilian passerines and Paradilepis caballeroi from Phalacrocorax in Mexico. Three Raillietina species share the same host, Dromaius novaehollandiae, and the same distribution, Australia, with accessions AY382312 through AY382314 and listed as unpublished. Oochoristica hemidactyli is recorded from Hemidactylus brookii in India with accession MK937582, and a separate row lists Hemidactylus brookii with accession MK937577 from the same reference, suggesting a possible labeling or formatting inconsistency because the host appears as a species entry. Overall, the dataset covers a wide host range and broad geography, but it provides identifiers and sources rather than sequence lengths or sampling counts, so representativeness cannot be inferred from this table alone.

Navigate back to Table 3.

## Table 4 Long description

The table lists cestode taxa included in a COI gene analysis, giving each species’ host, geographic distribution, GenBank accession identifier, and literature source. It includes a newly described Oochoristica turpanoeremiadis from the lizard Eremias roborowskii in China with two accessions, PX218524 and PX218525, reported in the current study. Human-associated records include Bertiella studeri from Sri Lanka and an unidentified Bertiella species from Poland, each with a single accession. Several genera are represented by multiple entries, such as Anonchotaenia from Brazil, Malaysia, and Iraq; Moniezia from Spain, India, and Japan; and Raillietina from Japan, Australia, Egypt, and Malaysia. Hosts span reptiles, humans, domestic animals, birds, and wild carnivores, with some host fields left blank. References vary between published studies, unpublished records, and entries noted as uploaded directly, so documentation detail is not consistent across rows.

Navigate back to Table 4.

## Table 5 Long description

The table lists cestode taxa included in an ITS1 through 5.8S through ITS2 phylogenetic dataset, giving each species, its host, geographic distribution, GenBank accession, and citation. Oochoristica turpanoeremiadis sp. nov. is represented by four accessions from Eremias roborowskii in China and is the only entry attributed to the current study. Oochoristica hemidactyli appears twice, both from Hemidactylus brookii in India, with two different GenBank IDs. Two Staphylocystis entries share the same host, Sorex araneus, and are both reported from America with references to Tkach and colleagues, 2013. Other taxa broaden host and region coverage, including Anoplocephala from gorilla in Rwanda and horse in Germany and the United Kingdom, Raillietina from emu in Australia, Mesocestoides from domestic cat in Germany, and Proteocephalus from squid in South Korea. Several records are marked unpublished or uploaded directly, so supporting publication details may be limited for those sequences.

Navigate back to Table 5.

## Table 6 Long description

The table compares hosts, infection sites, literature sources, and detailed body and reproductive measurements for seven Oochoristica tapeworm species. Body length ranges from 14.4 to 33.7 mm in O. whitfieldi and 15 to 20 mm in O. chalcidesi up to 57.6 mm in O. leonregagnonae and 65 mm in O. harschi, with O. hemidactyli at 49 to 53 mm. Proglottid counts vary from 23 in O. hemidactyli to 145 in O. harschi, with intermediate values such as 98 in O. leonregagnonae and 59 to 69 in O. chalcidesi. Scolex and sucker dimensions differ notably, including scolex width of 420 to 480 in O. chalcidesi versus 152 to 187 in O. hemidactyli, and sucker length reaching 190 to 230 in O. leonregagnonae. Segment measurements generally increase from immature to gravid proglottids, with gravid lengths up to 1200 to 3000 in O. leonregagnonae and 1450 to 2250 in O. whitfieldi. Reproductive traits also vary: testes counts range from 14 to 20 in O. harschi to 78 to 112 in O. leonregagnonae, and egg diameter spans 19 to 23 in O. whitfieldi up to 63 to 70 in O. chalcidesi. Hosts differ by species and infection site is typically intestine or small intestine. Values are mostly reported as ranges, some cells are missing, and O. harschi is reported as single fixed measurements, so direct comparisons should note uneven reporting detail.

Navigate back to Table 6.
