# Supplementary material for: Molecular characterization and phylogenetic placement of a new cestode, Oochoristica turpanoeremiadis sp. nov. (Cestoda: Cyclophyllidea: Linstowiidae), parasitizing Eremias roborowskii from Turpan Basin in China
Source: Parasitology. 2026 Jun 22;153(8):1000–13. doi: 10.1017/S0031182026102315 (PMC13735927; doi:10.1017/S0031182026102315)
Supplement: Supplementary file 1 [file S0031182026102315sup001.docx]

**Supplementary** **Table 1.** Uncorrected p-distances of the *18S* rRNA gene sequences of *Oochoristica turpanoeremiadis* sp. nov.

|  | 1 | 2 | 3 | 4 | 5 | 6 | 7 | 8 | 9 | 10 | 11 | 12 | 13 | 14 |
| --- | --- | --- | --- | --- | --- | --- | --- | --- | --- | --- | --- | --- | --- | --- |
| 1.*Oochoristica turpanoeremiadis* |  |  |  |  |  |  |  |  |  |  |  |  |  |  |
| 2.*Oochoristica turpanoeremiadis* | 0.0090 |  |  |  |  |  |  |  |  |  |  |  |  |  |
| 3.*Oochoristica hemidactyli* | 0.0678 | 0.0671 |  |  |  |  |  |  |  |  |  |  |  |  |
| 4.*Oochoristica hemidactyli* | 0.0678 | 0.0671 | 0.0000 |  |  |  |  |  |  |  |  |  |  |  |
| 5.*Anonchotaenia cf* | 0.1624 | 0.1632 | 0.1400 | 0.1400 |  |  |  |  |  |  |  |  |  |  |
| 6.*Anonchotaenia brasiliensis* | 0.1538 | 0.1546 | 0.1317 | 0.1317 | 0.0681 |  |  |  |  |  |  |  |  |  |
| 7.*Raillietina beveridgei* | 0.1280 | 0.1273 | 0.1141 | 0.1205 | 0.2103 | 0.1160 |  |  |  |  |  |  |  |  |
| 8.*Raillietina chiltoni* | 0.1286 | 0.1279 | 0.1172 | 0.1234 | 0.2137 | 0.1215 | 0.0442 |  |  |  |  |  |  |  |
| 9.*Raillietina dromaius* | 0.1210 | 0.1203 | 0.1109 | 0.1172 | 0.2116 | 0.1233 | 0.0442 | 0.0180 |  |  |  |  |  |  |
| 10.*Echinococcus canadensis* | 0.1538 | 0.1547 | 0.1422 | 0.1479 | 0.2281 | 0.1340 | 0.1276 | 0.1294 | 0.1251 |  |  |  |  |  |
| 11.*Echinococcus eguinus* | 0.1565 | 0.1574 | 0.1455 | 0.1511 | 0.2329 | 0.1391 | 0.1340 | 0.1348 | 0.1310 | 0.0187 |  |  |  |  |
| 12.*Taenia nwitchelli* | 0.1705 | 0.1712 | 0.1581 | 0.1633 | 0.2553 | 0.1689 | 0.1111 | 0.1177 | 0.1073 | 0.1284 | 0.1320 |  |  |  |
| 13.*Taenia martis* | 0.1744 | 0.1751 | 0.1638 | 0.1690 | 0.2496 | 0.1674 | 0.1134 | 0.1177 | 0.1068 | 0.1239 | 0.1271 | 0.0177 |  |  |
| 14.*Paradilepis caballeroi* | 0.1570 | 0.1579 | 0.1446 | 0.1505 | 0.2322 | 0.1413 | 0.1180 | 0.1179 | 0.1124 | 0.1426 | 0.1479 | 0.1307 | 0.1312 |  |
