# Supplementary material for: Molecular characterization and phylogenetic placement of a new cestode, Oochoristica turpanoeremiadis sp. nov. (Cestoda: Cyclophyllidea: Linstowiidae), parasitizing Eremias roborowskii from Turpan Basin in China
Source: Parasitology. 2026 Jun 22;153(8):1000–13. doi: 10.1017/S0031182026102315 (PMC13735927; doi:10.1017/S0031182026102315)
Supplement: Supplementary file 2 [file S0031182026102315sup002.docx]

**Supplementary** **Table 2.** Uncorrected p-distances (%) of the *COI* sequences of *Oochoristica turpanoeremiadis* sp. nov.

|  | 1 | 2 | 3 | 4 | 5 | 6 | 7 | 8 | 9 | 10 | 11 | 12 | 13 | 14 | 15 | 16 | 17 | 18 | 19 | 20 | 21 |
| --- | --- | --- | --- | --- | --- | --- | --- | --- | --- | --- | --- | --- | --- | --- | --- | --- | --- | --- | --- | --- | --- |
| 1.*Oochoristica turpanoeremiadis* |  |  |  |  |  |  |  |  |  |  |  |  |  |  |  |  |  |  |  |  |  |
| 2.*Oochoristica turpanoeremiadis* | 0.0000 |  |  |  |  |  |  |  |  |  |  |  |  |  |  |  |  |  |  |  |  |
| 3.*Beriella sp.* | 0.1627 | 0.1627 |  |  |  |  |  |  |  |  |  |  |  |  |  |  |  |  |  |  |  |
| 4.*Beriella studeri* | 0.1601 | 0.1601 | 0.0650 |  |  |  |  |  |  |  |  |  |  |  |  |  |  |  |  |  |  |
| 5.*Aviiellina sp.* | 0.2243 | 0.2220 | 0.2073 | 0.2047 |  |  |  |  |  |  |  |  |  |  |  |  |  |  |  |  |  |
| 6.*Aviiellina centripunctata* | 0.2105 | 0.2105 | 0.2117 | 0.2134 | 0.1287 |  |  |  |  |  |  |  |  |  |  |  |  |  |  |  |  |
| 7.*Anonchotaenia proIixa* | 0.1576 | 0.1553 | 0.1759 | 0.1549 | 0.2406 | 0.2076 |  |  |  |  |  |  |  |  |  |  |  |  |  |  |  |
| 8.*Anonchotaenia kornyushini* | 0.1620 | 0.1620 | 0.1496 | 0.1496 | 0.2496 | 0.2281 | 0.1475 |  |  |  |  |  |  |  |  |  |  |  |  |  |  |
| 9.*Anonchotaenia macrocephale* | 0.1788 | 0.1788 | 0.1785 | 0.1680 | 0.2335 | 0.2105 | 0.1444 | 0.1349 |  |  |  |  |  |  |  |  |  |  |  |  |  |
| 10.*Anonchotaenia globata* | 0.1704 | 0.1704 | 0.1477 | 0.1429 | 0.2185 | 0.2111 | 0.1370 | 0.1148 | 0.1370 |  |  |  |  |  |  |  |  |  |  |  |  |
| 11.*Mesocestoides sp.* | 0.1945 | 0.1922 | 0.1785 | 0.1864 | 0.2301 | 0.1842 | 0.1727 | 0.1681 | 0.1748 | 0.1370 |  |  |  |  |  |  |  |  |  |  |  |
| 12.*Mesocestoides litteratus* | 0.1894 | 0.1894 | 0.1877 | 0.1856 | 0.2247 | 0.2135 | 0.1717 | 0.1667 | 0.1843 | 0.1370 | 0.1263 |  |  |  |  |  |  |  |  |  |  |
| 13.*Moniezia expansa* | 0.1685 | 0.1685 | 0.1795 | 0.1829 | 0.2038 | 0.2018 | 0.1848 | 0.1848 | 0.1902 | 0.1704 | 0.1712 | 0.1929 |  |  |  |  |  |  |  |  |  |
| 14.*Moniezia sp.* | 0.1435 | 0.1435 | 0.1770 | 0.1667 | 0.2057 | 0.1770 | 0.1722 | 0.1962 | 0.1914 | 0.1579 | 0.1627 | 0.1722 | 0.1292 |  |  |  |  |  |  |  |  |
| 15.*Moniezia benedeni* | 0.1818 | 0.1813 | 0.1802 | 0.1526 | 0.2187 | 0.2135 | 0.1818 | 0.1978 | 0.1901 | 0.1852 | 0.2027 | 0.1910 | 0.1314 | 0.1005 |  |  |  |  |  |  |  |
| 16.*Moniezia sp.* | 0.1785 | 0.1762 | 0.1680 | 0.1444 | 0.2500 | 0.1959 | 0.1783 | 0.1849 | 0.1622 | 0.1704 | 0.1987 | 0.1843 | 0.1114 | 0.1196 | 0.0800 |  |  |  |  |  |  |
| 17.*Raillietina sp.* | 0.1731 | 0.1710 | 0.1828 | 0.1759 | 0.2399 | 0.2177 | 0.1858 | 0.1818 | 0.2031 | 0.1804 | 0.1884 | 0.2170 | 0.1988 | 0.1692 | 0.1944 | 0.2424 |  |  |  |  |  |
| 18.*Raillietina mitchelli* | 0.1738 | 0.1738 | 0.1864 | 0.1759 | 0.2381 | 0.1959 | 0.1643 | 0.1690 | 0.1881 | 0.1778 | 0.1810 | 0.1843 | 0.1984 | 0.1579 | 0.1950 | 0.2000 | 0.1766 |  |  |  |  |
| 19.*Raillietina hymenolepidoides* | 0.1884 | 0.1864 | 0.1969 | 0.1732 | 0.2426 | 0.2222 | 0.1667 | 0.1815 | 0.1685 | 0.1667 | 0.1838 | 0.1969 | 0.1984 | 0.1292 | 0.1937 | 0.1778 | 0.1696 | 0.1189 |  |  |  |
| 20.*Raillietina mahnerti* | 0.1901 | 0.1878 | 0.1785 | 0.1601 | 0.2388 | 0.2368 | 0.1781 | 0.1795 | 0.1745 | 0.1556 | 0.1915 | 0.1894 | 0.1957 | 0.1579 | 0.1841 | 0.1849 | 0.1838 | 0.1310 | 0.0833 |  |  |
| 21.*Taenia taeniaeformis* | 0.1742 | 0.1742 | 0.1689 | 0.1579 | 0.2449 | 0.2281 | 0.1793 | 0.1717 | 0.1742 | 0.1593 | 0.1995 | 0.1768 | 0.2011 | 0.2105 | 0.2219 | 0.2071 | 0.1923 | 0.2121 | 0.2276 | 0.2298 |  |
