# Supplementary material for: Molecular characterization and phylogenetic placement of a new cestode, Oochoristica turpanoeremiadis sp. nov. (Cestoda: Cyclophyllidea: Linstowiidae), parasitizing Eremias roborowskii from Turpan Basin in China
Source: Parasitology. 2026 Jun 22;153(8):1000–13. doi: 10.1017/S0031182026102315 (PMC13735927; doi:10.1017/S0031182026102315)
Supplement: Supplementary file 3 [file S0031182026102315sup003.docx]

**Supplementary** **Table 3.** Uncorrected p-distances of the *ITS1-5.8S-ITS2* sequences of *Oochoristica turpanoeremiadis* sp. nov.

|  | 1 | 2 | 3 | 4 | 5 | 6 | 7 | 8 | 9 | 10 | 11 | 12 | 13 | 14 |
| --- | --- | --- | --- | --- | --- | --- | --- | --- | --- | --- | --- | --- | --- | --- |
| 1.*Oochoristica turpanoeremiadis* |  |  |  |  |  |  |  |  |  |  |  |  |  |  |
| 2.*Oochoristica turpanoeremiadis* | 0.0092 |  |  |  |  |  |  |  |  |  |  |  |  |  |
| 3.*Oochoristica turpanoeremiadis* | 0.0040 | 0.0104 |  |  |  |  |  |  |  |  |  |  |  |  |
| 4.*Oochoristica turpanoeremiadis* | 0.0035 | 0.0081 | 0.0040 |  |  |  |  |  |  |  |  |  |  |  |
| 5.*Oochoristica hemidactyli* | 0.2744 | 0.2750 | 0.2742 | 0.2747 |  |  |  |  |  |  |  |  |  |  |
| 6.*Oochoristica hemidactyli* | 0.2750 | 0.2756 | 0.2748 | 0.2753 | 0.0000 |  |  |  |  |  |  |  |  |  |
| 7.*Proteocephalus sp.* | 0.5213 | 0.5222 | 0.5222 | 0.5222 | 0.4940 | 0.4929 |  |  |  |  |  |  |  |  |
| 8.*Anoplocephala gorillae* | 0.5018 | 0.5012 | 0.5012 | 0.5003 | 0.4638 | 0.4619 | 0.4858 |  |  |  |  |  |  |  |
| 9.*Anoplocephala perfoliata* | 0.5042 | 0.5030 | 0.5036 | 0.5027 | 0.4636 | 0.4600 | 0.4771 | 0.0446 |  |  |  |  |  |  |
| 10.*Staphylocystis sp.* | 0.5098 | 0.5081 | 0.5088 | 0.5074 | 0.4670 | 0.4639 | 0.4601 | 0.2713 | 0.2473 |  |  |  |  |  |
| 11.*Staphylocystis schilleri* | 0.5095 | 0.5078 | 0.5085 | 0.5071 | 0.4665 | 0.4634 | 0.4595 | 0.2721 | 0.2481 | 0.0011 |  |  |  |  |
| 12.*Raillietina dromaius* | 0.6043 | 0.6058 | 0.6058 | 0.6054 | 0.6130 | 0.6130 | 0.6526 | 0.6175 | 0.6285 | 0.6224 | 0.6250 |  |  |  |
| 13.*Raillietina beveridgei* | 0.6193 | 0.6206 | 0.6206 | 0.6203 | 0.6092 | 0.6092 | 0.6354 | 0.6075 | 0.6260 | 0.6234 | 0.6258 | 0.2561 |  |  |
| 14.*Mesocestoides litteratus* | 0.5388 | 0.5384 | 0.5392 | 0.5388 | 0.5327 | 0.5294 | 0.3228 | 0.5247 | 0.5212 | 0.5000 | 0.5000 | 0.6305 | 0.6310 |  |
